# Lidocaine-Loaded Solid Lipid Microparticles (SLMPs) Produced from Gas-Saturated Solutions for Wound Applications

**DOI:** 10.3390/pharmaceutics12090870

**Published:** 2020-09-12

**Authors:** Clara López-Iglesias, Cristina Quílez, Joana Barros, Diego Velasco, Carmen Alvarez-Lorenzo, José L. Jorcano, Fernando J. Monteiro, Carlos A. García-González

**Affiliations:** 1 Department of Pharmacology, Pharmacy and Pharmaceutical Technology, I+D Farma group (GI-1645), Faculty of Pharmacy, Agrupación Estratégica de Materiales (AeMAT) and Health Research Institute of Santiago de Compostela (IDIS), Universidade de Santiago de Compostela, 15782 Santiago de Compostela, Spain; clara.lopez.iglesias@rai.usc.es (C.L.-I.); carmen.alvarez.lorenzo@usc.es (C.A.-L.); 2 Department of Bioengineering and Aerospace Engineering, Universidad Carlos III de Madrid (UC3M), 28911 Leganés (Madrid), Spain; cquilez@ing.uc3m.es (C.Q.); divelasc@ing.uc3m.es (D.V.); jjorcano@ing.uc3m.es (J.L.J.); 3 Instituto de Investigação e Inovação em Saúde da Universidade do Porto (i3S), Instituto de Engenharia Biomédica (INEB), Faculdade de Engenharia (FEUP), Universidade do Porto, 4200-135 Porto, Portugal; joana.barros@ineb.up.pt (J.B.); fjmont@fe.up.pt (F.J.M.)

**Keywords:** SLMPs, drug delivery, lidocaine HCl, 3D-bioprinting, PGSS technique, wound healing, pain

## Abstract

The delivery of bioactive agents using active wound dressings for the management of pain and infections offers improved performances in the treatment of wound complications. In this work, solid lipid microparticles (SLMPs) loaded with lidocaine hydrochloride (LID) were processed and the formulation was evaluated regarding its ability to deliver the drug at the wound site and through the skin barrier. The SLMPs of glyceryl monostearate (GMS) were prepared with different LID contents (0, 1, 2, 4, and 10 wt.%) using the solvent-free and one-step PGSS (Particles from Gas-Saturated Solutions) technique. PGSS exploits the use of supercritical CO_2_ (scCO_2_) as a plasticizer for lipids and as pressurizing agent for the atomization of particles. The SLMPs were characterized in terms of shape, size, and morphology (SEM), physicochemical properties (ATR-IR, XRD), and drug content and release behavior. An in vitro test for the evaluation of the influence of the wound environment on the LID release rate from SLMPs was studied using different bioengineered human skin substitutes obtained by 3D-bioprinting. Finally, the antimicrobial activity of the SLMPs was evaluated against three relevant bacteria in wound infections (*Escherichia coli*, *Staphylococcus aureus,* and *Pseudomonas aeruginosa*). SLMPs processed with 10 wt.% of LID showed a remarkable performance to provide effective doses for pain relief and preventive infection effects.

## 1. Introduction

Pain is one of the most debilitating symptoms of patients suffering from chronic and post-surgical wounds, especially during wound debridement and dressing exchange [1]. As an example, ca. 80% of patients suffering from venous leg ulcers report acute or chronic wound pain, which causes discomfort, stress, anxiety and, overall, poor quality of life [2,3,4,5]. Local anesthetics, and lidocaine hydrochloride (LID) in particular, have been used for decades to reduce wound pain (e.g., in post-operative analgesia) without hampering the physiological scarring process [6,7]. The mechanism of action of LID is the specific interaction and blockage of sodium channels of the sensorial neurons [8]. Through this LID–neuron interaction, when the nerve impulse arrives to the cell and the cell membrane is depolarized, sodium cations are unable to enter the neuron and the nerve impulse transmission is stopped. Furthermore, LID also has certain antimicrobial activity against common bacteria in nosocomial and surgical wound infections (e.g., *Enterococcus faecalis*, *Escherichia coli*, *Staphylococcus aureus,* and *Pseudomonas aeruginosa*), so its local infusion may also prevent bacterial colonization in post-operative wounds [9,10].

Particulate systems like micro- and nanoparticles are of increasing interest for drug delivery to the wound site [11,12]. The use of micron- and nano-sized systems based on organic and inorganic materials to deliver bioactive agents improves their efficacy and safety, and increases their physicochemical stability by protecting them from the environment (e.g., enzymatic degradation) [13,14,15]. Microparticles are often preferred for local drug delivery since they remain in the application site while providing a sustained drug release [15]. Lipids are regarded as advantageous components of particulate matrices for wound care, since they can mimic the natural components of skin and modulate the drug release [16,17,18,19,20]. Namely, solid lipid microparticles (SLMPs) usually present higher physicochemical stability and are easier to sterilize and to scale-up than other lipid-based drug carriers like liposomes [21]. SLMPs have not been extensively studied for dermal and wound delivery, but have shown as potential candidates for topical and transdermal delivery of several drugs like econazole nitrate, testosterone, and tetracycline [22,23].

SLMPs processing usually requires the use of organic solvents and emulsifying agents, but novel processing strategies are arising to improve environmental and economic aspects. Supercritical CO_2_ (scCO_2_) has emerged as a processing tool for the development of new, green, and inexpensive techniques that require mild conditions and avoid/mitigate organic solvents [14,24,25,26]. Namely, the PGSS (Particles from Gas-Saturated Solutions) technique is based on the dissolution of scCO_2_ in a molten substance, followed by a rapid expansion through a nozzle of the molten substance that causes solidification and precipitation of solid microparticles [27,28,29,30]. Unlike other compressed fluids, scCO_2_ can interact with the processed substances at a molecular level decreasing their melting temperature, which may be advantageous for the scale-up of the process in terms of energy and cost optimization [28,31]. The processing of SLMPs using the PGSS technique avoids the use of solvents or washing steps. High loading efficiencies of bioactive agents (insulin, recombinant human growth hormone, ibuprofen, ketoprofen) are commonly obtained using this technique [32,33,34,35,36]. The advantageous features of PGSS technique can be herein exploited for the processing of SLMPs loaded with LID. These SLMPs can lead to a more prolonged release of LID and a longer duration of action than commercial LID-containing ointments, gels, and solutions. LID-loaded particles may thus adapt to the demands of the pharmacological action site (wound) where the damaged tissue accelerates drug permeation due to the absence of epidermis and the modified LID release also reduces the drug side effects of systemic absorption.

Drug permeability and toxicity in vitro test alternatives to the use of human or animal native skin are pursued to evaluate the performance of topical formulations, since these in vivo practices are constrained by economic, ethical, and practical reasons or even by regulation in the case of cosmetics [37,38]. Following the 3Rs principles and with the rise of the biomedical engineering discipline, several in vitro artificial skin models with attractive versatility and reproducibility are being commercialized or under development to reflect the three-dimensional environment of human native skin and the in vivo drug release conditions through the skin barrier [39]. 3D-bioprinting has emerged as an ideal technology to design more complex artificial skin models under automatized and standardized protocols able to mimic the native skin in a more reproducible manner [40]. The versatility of 3D-bioprinting has high translational and clinical relevance, since this technology can be exploited to produce not only full-thickness healthy skin, but also damaged skin under different pathological situations like acute and chronic wounds [41].

In this work, the PGSS technique was implemented for the first time to produce SLMPs for specific wound delivery applications in pain and infection treatment. The SLMPs consisted on a matrix of glyceryl monostearate (GMS) loaded with LID at different contents (0, 1, 2, 4, and 10 wt.%) in an attempt to get formulations in the typical LID topical dosage range (0.5–5 wt.%) [42,43]. To the best of our knowledge, it is the first time that SLMPs loaded with LID were processed by the PGSS technique. Obtained SLMPs were characterized in terms of physicochemical properties and drug content. Drug release behavior of LID from the SLMPs was evaluated using Franz cells and bioengineered skin substitutes with and without epidermis to evaluate the transport and release of LID through the intact skin and open wound skin tissues. Finally, the antimicrobial activity of LID released from the SLMPs against susceptible bacterial strains commonly present in infected wounds (*E. coli*, *S. aureus,* and *P. aeruginosa*) was assessed.

## 2. Materials and Methods

### 2.1. Materials

Kolliwax^®^ GMS II (glycerolmonostearate 40-55 type II, powder) was supplied by BASF (Ludwigshafen am Rhein, Germany). Lidocaine HCl monohidrate (≥99% purity) was purchased from Sigma-Aldrich (Saint Louis, MO, USA). CO_2_ (99.8% purity) was supplied by Praxair (Madrid, Spain). Glacial acetic acid (purity > 99.0%) and acetonitrile (HPLC grade, purity > 99.9%) were purchased from Scharlau (Barcelona, Spain). NaOH (98.0% purity) was obtained from Panreac (Barcelona, Spain). Water was purified using reverse osmosis (resistivity > 18 MΩ·cm, Milli-Q, Millipore^®^, Madrid, Spain). Mueller-Hinton agar and broth were supplied by Merck Millipore (Darmstadt, Germany) and were used to perform the antimicrobial assays.

Human primary fibroblasts (hFBs) and human primary keratinocytes (hKCs) obtained from skin biopsies of healthy donors were obtained from the collections of biological samples of human origin, which are registered in the Registro Nacional de Biobancos para Investigación Biomédica del Instituto de Salud Carlos III with reference C.0002961. This collection is located in the Centro Comunitario de Sangre y Tejidos (CCST, Oviedo, Spain) and in the CIEMAT. Fresh frozen human plasma was provided by a local blood bank (Banco de Sangre del Centro Comunitario de Transfusión del Principado de Asturias, Oviedo, Spain), and was obtained following the standards of the American Association of Blood Banks [44].

### 2.2. SLMPs Production by the PGSS Technique

Mixtures (6 g) of GMS manually mixed with a LID content of 0, 1, 2, 4, and 10 wt.% were loaded into a 300-mL high-pressure saturator (Eurotechnica GmbH, Bargteheide, Germany). After heating the saturator to 67 °C, CO_2_ entered the equipment at a constant flow of 7 g/min until a pressure of 170 bar was reached. After 1 h of contact of the compressed CO_2_ with the molten lipid and drug under agitation at 400 rpm, the system was depressurized by the opening of a valve placed at the bottom of the saturator. The mixture was thus sprayed through a nozzle of 1 mm of diameter, and the fast depressurization caused the precipitation of the lipid-based formulation into solid microparticles inside a precipitator vessel of 2.7 L. SLMPs were then collected and kept in 50-mL Falcon tubes for further use. The SLMPs were denoted as GMS-LID*x*, *x* being the theoretical content of LID expressed as wt.%.

### 2.3. Analysis of the Morphology, Size, and Surface of the Particles

Images of the GMS particles processed by the PGSS technique were obtained by scanning electron microscopy (SEM, EVO LS 15, Zeiss, Oberkochen, Germany) to evaluate the morphology, size, homogeneity, and surface of the particles. Prior to imaging, SLMPs were sputtered-coated (Q150T S/E/ES, Quorum Technologies, Lewes, UK) with a layer of 10 nm of iridium to improve the contrast. Particle sizes were measured by image analysis using ImageJ v1.49 software (National Institutes of Health, Bethesda, MD, USA).

### 2.4. Physicochemical Characterization

Raw GMS powder, raw LID powder, GMS-LID0 and GMS-LID4 particles were analyzed by Attenuated Total Reflectance/Fourier-Transform Infrared Spectroscopy (ATR/FT-IR). A Gladi-ATR accessory equipped with a diamond crystal (Pike, Madison, WI, USA) was used. Spectra were collected in the mid-IR spectrum range (400–4000 cm^−1^) using 32 scans at a resolution of 2 cm^−1^. Crystallinity of the raw materials and GMS-LID0 and GMS-LID4 particles obtained by the PGSS technique were studied by X-ray diffraction (XRD, PW-1710, Philips, Eindhoven, The Netherlands) in the 2–50° 2θ-range using a 0.02° step and CuKα_1_ radiation. The raw materials, GMS-LID0 and GMS-LID4 were also analyzed using differential scanning calorimetry (DSC, DSC-Q100, TA Instruments; New Castle, DE, USA). Samples were weighted in an aluminum pan, cooled down from room temperature to 0 °C, and then heated up to 150 °C at a rate of 5 °C/min.

### 2.5. Determination of LID Encapsulation Efficiency

The encapsulation efficiency (EE) was evaluated by dispersing 26 mg of GMS-LID1, GMS-LID2, GMS-LID4, and GMS-LID10 particles in 10 mL of PBS pH 7.4 under agitation (700 rpm). The suspensions were heated at 70 °C for 4 h until microparticles were molten and the entrapped drug was fully dissolved. Afterwards, the suspensions were centrifuged and cooled down (4000 rpm, 20 °C) and 1 mL of the supernatant was extracted and filtered with syringe filters (hydrophilic PTFE, 0.22 μm, Scharlau, Barcelona, Spain) for HPLC measurements.

LID concentration was measured by HPLC (JASCO Spain, Madrid, Spain) using a C18 column (Symmetry^®^, 5 μm, 3.9 × 150 mm, Waters, Milford, MA, USA) with isocratic elution [45]. The mobile phase consisted of acetonitrile:acetic acid 5% (pH 3.4 adjusted with 1 M NaOH solution) 15:85 (*v/v*). Flow was kept at 1 mL/min and temperature at 30 °C. LID had a retention time of 4.5 min, and the absorbance was measured at the wavelength of 262 nm. The calibration curve was carried out in the 1–100 μg/mL range (*R*^2^ = 0.9995).

The encapsulation efficiency (EE) was calculated following the equation (Equation (1)):

(1) $$\mathrm{EE}=\frac{w_{P}}{w_{T}}\times 100$$

where *w_P_* is the amount of LID contained in the SLMPs measured by HPLC (in mg LID per mg of SLMPs) and *w_T_* is the initial amount of LID added for PGSS processing (in mg LID per mg of GMS-LID mixture).

### 2.6. LID Release Tests from SLMPs

The release of LID from raw LID, GMS-LID1, GMS-LID2, GMS-LID4, and GMS-LID10 particles was performed in Franz diffusion cells consisting of a donor and a receptor compartment separated by a 0.45 μm cellulose nitrate membrane filter (Whatman, Little Chalfont, UK). Franz cells were kept in an orbital shaker (VWR^®^ Incubating Mini Shaker, VWR, Chester, PA, USA) at 37 °C under agitation at 100 rpm. The volume of the receptor compartment was 6 mL and the surface available for diffusion was ca. 0.8 cm^2^. The receptor compartment was filled with PBS pH 7.4 medium, and 25 mg of particles were placed in the donor compartment with 0.5 mL of PBS medium. Then, 1 mL-aliquots were taken every hour for the first 8 h and then after 24 h, and the same volume was replaced with fresh PBS solution. Samples were filtered with syringe filters (hydrophilic PTFE, 0.22 μm, Scharlau, Barcelona, Spain) and measured by HPLC (see Section 2.5). The release of LID from SLMPs was compared to the dissolution of 3 mg of pure LID, carried out using the same experimental set-up.

LID release profiles of the drug were fitted to the first-order (Equation (2)) and the first-order with lag time (Equation (3)) release kinetics models:

(2) $$\%\;\mathrm{LID}\;\mathrm{released}\;=\;100\times (1-e^{-k_{1}\times t})$$

(3) $$\%\;\mathrm{LID}\;\mathrm{released}\;=\;100\times (1-e^{-k_{2}\times \left(t-t_{lag}\right)})$$

where *k*_1_ and *k*_2_ are the kinetic coefficients, *t* is the elapsed release time, and *t_lag_* is the lag time.

### 2.7. Preparation of Bioprinted Human Skin Equivalents

Bioprinted human skin substitutes were generated following a previously developed method [46]. Briefly, two cellular layers were printed: a dermal layer, formed by a plasma-derived fibrin matrix populated with hBFs, and an epidermal layer formed by hKCs seeded on top. Cell cultures were printed on polyethylene terephthalate (PET) cellular inserts (Falcon 353102, 4.2 cm^2^ area, 1 μm pore diameter; Thermo Fischer Scientific, Waltham, MA, USA) in a 6-well culture plate (4.15 cm^2^, Corning Costar Corp., Cambridge, MA, USA) and were allowed to differentiate at the air–liquid interface for 17 days at 37 °C in a CO_2_ incubator in hKCs medium containing 0.5% FBS and 50 μg of ascorbic acid as differentiating medium (Figure S1A). The medium was changed every three days.

### 2.8. LID Release Tests through Bioprinted Human Skin Equivalents

**Figure 1:**
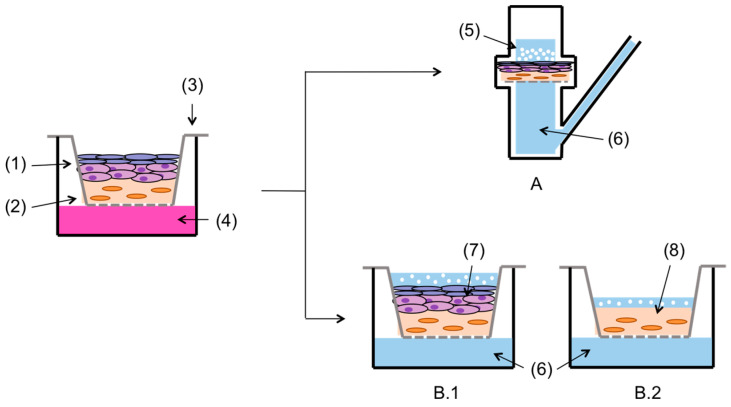
Full thickness skin substitute consisting of epidermal (1) and dermal (2) tissues was cultured on cellular inserts (3) using human primary keratinocytes (hKCs) culture medium (4). For permeation tests, the base of the cellular insert with full thickness skin was placed as separator membrane in Franz cells (**A**), usingraw lidocaine hydrochloride (LID) or drug-loaded GMS-LID4 particles (5) in the donor compartment. The release medium was PBS pH 7.4 (6). Another part of the experiment tested the permeation of LID from GMS-LID4 particles directly in the cellular inserts, using (**B.1**) either full thickness skin substitute (7) containing the keratinocyte layer, (**B.2**) or dermal tissue (8) without epidermis as membrane.

Firstly, the diffusion of LID dissolved in PBS from pure LID powder and the GMS-LID4 microparticles through the intact bioengineered skin equivalent, i.e., with epidermal and dermal layers were compared. The bioengineered skin equivalents were collected (Figure S1B) and cut with a scalpel in circles of 2 cm of diameter, and used as membranes in 6-mL Franz diffusion cells (Figure 1A). Release tests and LID measurements by HPLC were carried out in four replicates and using the same protocols as in Section 2.5.

A second part of the LID release tests was carried out to compare the diffusion of LID from SLMPs through intact bioengineered skin equivalents (Figure 1(B.1)) and the same skin equivalents without epidermis (only dermis, Figure 1(B.2)) to mimic an open skin wound. Using the bioengineered skin equivalents in the cellular insert, the epidermis was removed with the help of precision forceps. The experiment was carried out in triplicate and directly in the cellular inserts adapting a protocol from the literature [47]. GMS-LID4 particles were added to each insert along with 0.5 mL of PBS to facilitate their humectation. The 6-well culture plate was filled with 10 mL of PBS pH 7.4 as the receptor medium. The 1-mL aliquots were taken at the same selected times, filtered with syringe filters (hydrophilic PTFE, 0.22 μm pore diameter; Scharlau, Barcelona, Spain), and measured by HPLC (see Section 2.5). The extracted volume was replaced with equal volumes of fresh PBS medium.

Histological analysis of the bioengineered skin equivalents was performed before and after (24 h) the drug release experiments to assess their integrity and whether the particles penetrated them. Skin samples were fixed in 3.7% buffered formaldehyde for 24 h and embedded in paraffin. Then, 5-μm cross-sections were dewaxed, rehydrated, and stained with hematoxylin-eosin (H&E).

### 2.9. Antimicrobial Tests

The antimicrobial activity of LID was assessed against susceptible bacterial strains commonly present in infected wounds: *E. coli* 25922, *S. aureus* 33591, and *P. aeruginosa* PA01. Susceptibility tests to LID were performed by determination of the minimum inhibitory concentration (MIC) and minimum bactericidal concentration (MBC) of LID powder, according to the CLSI standards broth microdilution procedure. Furthermore, the antimicrobial activity of LID released from 40 mg of GMS-LID10 particles in 5 mL of PBS medium after 6 h was evaluated by quantifying the cultivable bacteria.

#### 2.9.1. Minimum Inhibitory Concentration

Bacteria were grown overnight at 37 °C in Mueller-Hinton Broth (MHB) at 150 rpm. After incubation, a bacterial suspension was adjusted to ca. 10^6^ colony forming units (CFU/mL). Subsequently, 100 µL of bacterial suspension and 100 µL of LID at different concentrations (39,400, 19,700, 9850, 4925, 2463, and 1231 µg/mL) were placed in 96-well microplates and incubated for 24 h at 37 °C and 150 rpm. Bacterial growth (turbidity) was evaluated measuring absorbance at 640 nm in a microplate reader. All tests were performed in quintuplicate with two repeats. MICs were expressed in terms of µg/mL [48]. Two controls were used to perform the antimicrobial tests: a positive control representing the bacterial growth without LID (0 µg/mL) and a negative control representing the absorbance of medium without bacteria.

#### 2.9.2. Minimum Bactericidal Concentration

Bacteria were grown overnight in MHB at 37 °C and 150 rpm. After incubation, a bacterial suspension was adjusted to ca. 10^6^ colony forming units (CFU/mL). Then, 100 µL of bacterial suspension and 100 µL of LID (at the same concentrations as for the MIC determination) were placed in 96-well microplates and incubated for 24 h at 37 °C and 150 rpm. Subsequently, cultivable bacteria were quantified by colony counting in Mueller-Hinton Agar (MHA). All experiments were performed in quintuplicate with two repeats. MBCs were expressed in terms of µg/mL [48]. Two controls were used to perform the antimicrobial tests: a positive control representing the bacterial growth without LID (0 µg/mL) and a negative control representing the initial bacterial concentration.

#### 2.9.3. Antimicrobial Activity of LID Released from GMS

Bacteria were grown overnight in MHB at 37 °C and 150 rpm and a bacterial suspension of ca. 10^7^ cells/mL. GMS-LID10 particles (40 mg) were dispersed in 5 mL of PBS pH 7.4. Then, 180 µL of PBS with LID released from GMS-LID10 particles and 20 µL of bacterial suspension were placed (five for each condition) in 96-well plates. After 24 h incubation at 37 °C and 150 rpm, the cultivable bacteria were quantified by the colony-forming units (CFUs) method. Bacterial density (in log_10_ CFUs/mL) was plotted according to each condition. Bacterial growth without LID was used as control. All experiments were performed in quintuplicate with two repeats.

### 2.10. Statistical Analysis

Results were reported as mean ± standard deviation. Statistical analysis of the antimicrobial tests was performed using one-way analysis of variance (ANOVA) test followed by post-hoc Tukey HSD multiple comparison test (IBM^®^ SPSS^®^ Statistics v.22.0, Armonk, NY, USA), which was used to determine the significant difference (*p*).

## 3. Results and Discussion

### 3.1. Morphological and Physicochemical Properties of the SLMPs

**Figure 2:**
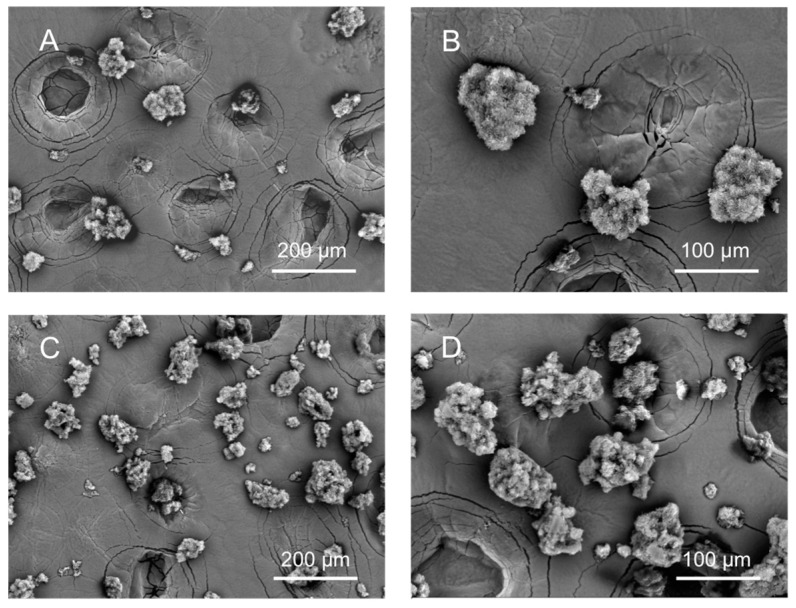
Scanning electron microscopy (SEM) images of (**A**,**B**) GMS-LID0 and (**C**,**D**) GMS-LID4 particles. The processing of GMS with LID did not influence the overall appearance and shape of the particles.

Obtained GMS particles were fine, white, and free-flowing powder. The conditions of pressure and temperature for the processing of GMS particles containing LID were selected above the melting point of GMS so they would provide good process ability. Thus, the SEM images showed an open and porous structure, caused by the escape of CO_2_ from the molten lipid and sudden solidification upon depressurization (Figure 2). The shape of the particles was non-spherical and particle diameter was in the 20–120 μm range with a mean of 114 µm, a standard deviation of 39 µm, and a PDI of 0.12. The non-sphericity of lipid particles using the PGSS technique is also observed using other SLMPs processing technologies like spray drying and this morphology can influence the drug release behavior due to shorter drug diffusion pathways [49,50]. The processing of GMS together with LID did not influence the overall appearance of the particles.

**Figure 3:**
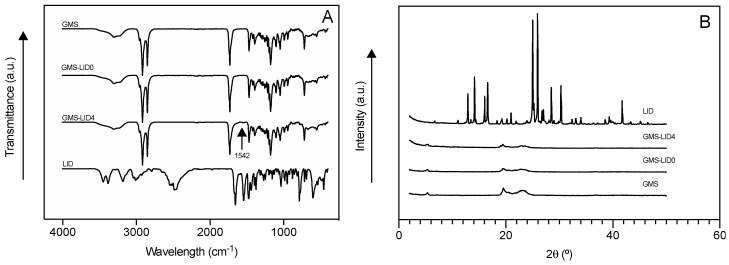
(**A**) Attenuated Total Reflectance/Fourier-Transform Infrared Spectroscopy (ATR/FT-IR) spectra and (**B**) X-ray diffraction (XRD) patterns of pure GMS, solid lipid microparticles (SLMPs) of GMS processed by the Particles from Gas-Saturated Solutions (PGSS) technique (GMS-LID0), SLMPs loaded with LID (GMS-LID4), and pure LID powder.

**Figure 4:**
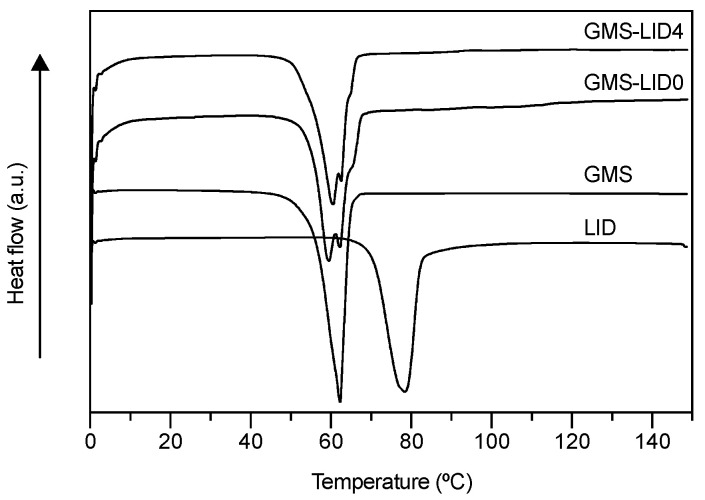
Differential scanning calorimetry (DSC) thermograms of the raw materials, GMS-LID0 and GMS-LID4 particles. No peaks corresponding to crystalline LID were appreciated in the GMS-LID4 particles.

ATR/FT-IR spectroscopic studies confirmed the presence of LID in the GMS-based particles (Figure 3A). The spectrum of GMS-LID4 particles showed a small band at the wavelength of 1542 cm^−1^, corresponding to the characteristic band of C=O carbonyl group stretching mode of LID [51]. Spectra of pure GMS powder and GMS processed by the PGSS technique were practically identical, proving that the processing with scCO_2_ did not alter the chemical structure of the GMS. The XRD analysis of the GMS-LID4 powder (Figure 3B) did not show the characteristic sharp peaks of crystalline LID, suggesting the change of the drug from the crystalline to the amorphous state upon processing with the PGSS technique. The absence of crystalline LID was also confirmed by the DSC analysis (Figure 4). No peaks corresponding to crystalline LID were observed in the thermogram of the GMS-LID4 particles. The main thermal events obtained from the DSC thermograms after peak integration are summarized in Table 1.

### 3.2. LID Encapsulation Efficiency

Initial LID contents for SLMPs processing were set in the 1–10 wt.% range taking into account the usual LID concentrations in topical LID formulations for pain treatment (0.5–5 wt.%) [42,43] and the high encapsulation efficiencies usually obtained by PGSS technology. Accordingly, LID encapsulation efficiencies for GMS-LID1, GMS-LID2, GMS-LID4, and GMS-LID10 particles were 69.6 ± 4.5%, 59.7 ± 13.6%, 79.0 ± 4.0%, and 75.1 ± 0.9%, respectively. These values correlated well with the encapsulation efficiency of other studies using the PGSS technique for the loading of drugs into microparticles [27], although it is the first time LID is incorporated. CO_2_ at supercritical conditions can act as a solvent for the drug, so drug loss could take place by the partial dissolution of the drug in scCO_2_ and its deposition on the walls of the equipment after depressurization [52]. Unlike lidocaine, the hydrochloride salt has no measurable solubility in scCO_2_ at temperatures up to 45 °C [53]. The solubility of LID in scCO_2_ may increase at higher temperatures, as it occurs with other anesthetic drugs. Nevertheless, high drug encapsulation efficiencies were herein obtained and related to the solvent-free processing of the particles and absence of downstream steps.

LID content in the different SLMPs processed by PGSS (GMS-LID1, GMS-LID2, GMS-LID4, and GMS-LID10) was 0.7 ± 0.0, 1.2 ± 0.3, 3.2 ± 0.2, and 7.5 ± 0.2 wt.%, respectively. The drug contents obtained in the SLMPs are thus compatible with formulations for local drug administration where recommended LID doses are 5–300 mg [42,43].

### 3.3. LID Release Tests

**Figure 5:**
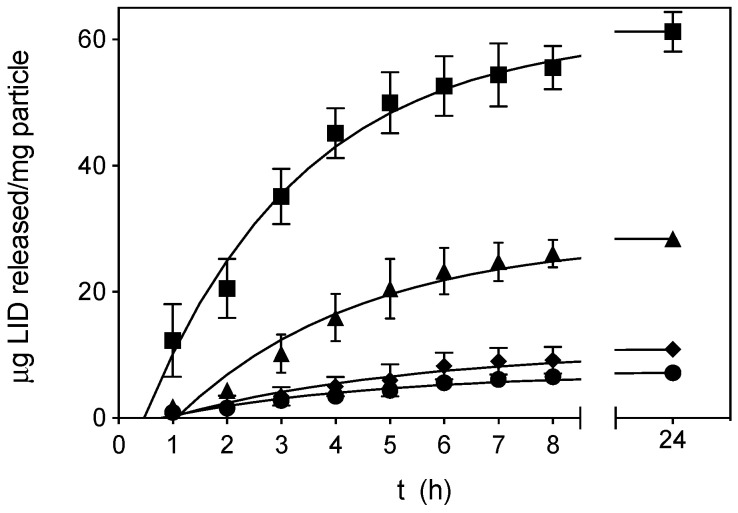
Release data of GMS-LID1 (circles), GMS-LID2 (diamonds), GMS-LID4 (triangles), and GMS-LID10 (squares) particles in PBS pH 7.4 at 37 °C and 100 rpm. Lines represent the fittings to a first-order kinetic release with lag time model (Equation (3)).

SLMPs were suspended in PBS medium at the donor compartment of the Franz diffusion cells, forming a thin layer. A sustained release of LID from the SLMPs was obtained (Figure 5), being at least 50% of the drug payload released after 4 h and the complete release attained after 24 h. The release profile was noticeably delayed with respect to the dissolution profile of pure LID in Franz cells (Figure S2), where more than 85% of the initial amount of LID was quantified after only 15 min, and the complete dissolution took place after 1 h. LID solutions used clinically for local infusion by subcutaneous injection present a duration of action of 0.5–2 h, normally keeping their effect only during a surgical procedure [54]. SLMPs providing a controlled release of the anesthetic agent could be useful to maintain local anesthesia for longer times, during the first stage of wound healing.

The LID release profile of GMS-LID1, GMS-LID2, GMS-LID4, and GMS-LID10 particles fitted well to a first-order kinetic model (Table 2). The first-order release occurs when the drug release rate is dependent on its concentration. The amount of drug released from the matrix is proportional to the amount remaining, so it tends to decrease with time [55]. The release profile fitted better to a first-order with lag time kinetic model. The LID dissolution in aqueous media is highly dependent on the hydration of the microparticles, since LID is a hydrophilic drug included within a lipid matrix. The hydrophobic nature of the GMS should delay this hydration and explains the lag times between 0.8 and 1.1 h. Once dissolved, LID release is conditioned by the diffusion of the drug through the lipid matrix.

### 3.4. LID Permeation through Bioengineered Skin Substitutes

Topical anesthetics like LID block reversibly the free nerve-ending conductions in the dermis and mucosa. This process can be limited by drug diffusion through the epidermis, since in healthy skin the drug has to pass through the stratum corneum to reach dermal tissue at therapeutic concentrations [56]. The versatility of 3D-bioprinting was exploited in this work to produce different types of skin substitutes (full-thickness healthy skin and dermal layer tissue) to study the effect of epidermis barrier on LID diffusion and to mimic the open wound skin tissue.

**Figure 6:**
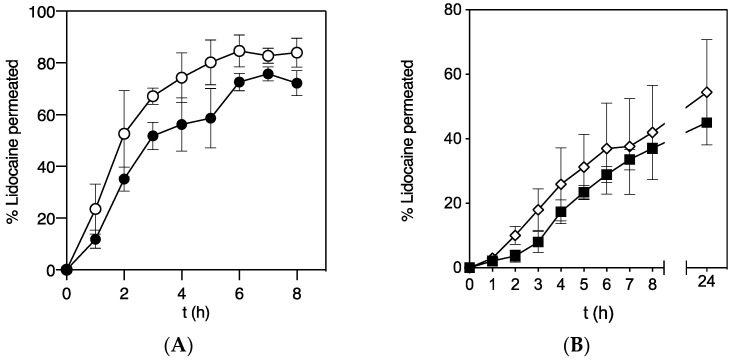
Permeability of LID through a bioengineered skin substitute comparing (**A**) the permeability of LID released from GMS-LID4 particles (squares) and LID powder (diamonds) in Franz cell experiments (*n* = 4), and (**B**) the permeability of LID released from GMS-LID4 particles through complete skin tissue (epidermal and dermal layers, black circles) and dermal layer (white circles) (*n* = 3).

The penetration of LID through a bioprinted skin equivalent based on two cellular layers, representing the dermis (lower layer) and the epidermis (upper layer) was thus firstly studied. The penetration of LID from GMS-LID4 particles and pure LID powder was compared using Franz cells (Figure 6A). Penetration of pure LID powder dissolved in the PBS medium was faster than LID released from GMS-LID4 particles. Probably, the release of LID from the particles acted as the limiting step, so LID firstly dissolved from the particles into the PBS medium and then passed through the membrane. Furthermore, LID powder dissolved very fast in the donor compartment and it is expected that the drug permeates faster at a higher concentration gradient between the donor and the receptor compartment.

The penetration of LID released from the particles through a full thickness skin substitute and through a dermal layer alone was compared (Figure 6B). This study was carried out using a 3D-tissue model with skin substitutive membranes prepared in inserts and located in multi-well plates since the dermal layer was very fragile and its removal from the insert compromised the integrity of the skin equivalents. This method was also advantageous to get a more compact setup and to facilitate the run of multiple samples in parallel [47]. The dermal layer is highly hydrated, so LID dissolved in PBS diffused faster through the dermis tissue. A higher percentage of permeated LID was detected in this experiment using cellular inserts with respect to the studies in the Franz cell experiments (Figure 6 and Figure S2). In accordance to the Fick’s second diffusion law, the higher drug permeation to the receptor compartment obtained with the cellular inserts was attributed to the much larger effective diffusion area (4.2 cm^2^) with respect to the Franz cells (0.8 cm^2^). However, when the LID permeation values were normalized per surface area, results showed that LID contained in the GMS particles permeated faster through the full thickness skin substitute in the Franz cells (0.37 mg LID/cm^2^ after 8h) than in the cellular inserts (0.14 mg LID/cm^2^ after 8 h). This difference could be attributed to a possible damage of the skin equivalent membranes during cutting and transfer prior to the Franz cell tests, or to a reduced actual effective diffusion area for LID permeation in the cellular inserts tests due to the presence of the supporting PET porous membrane of the commercial inserts located just below the skin substitute membrane (Figure 1B).

**Figure 7:**
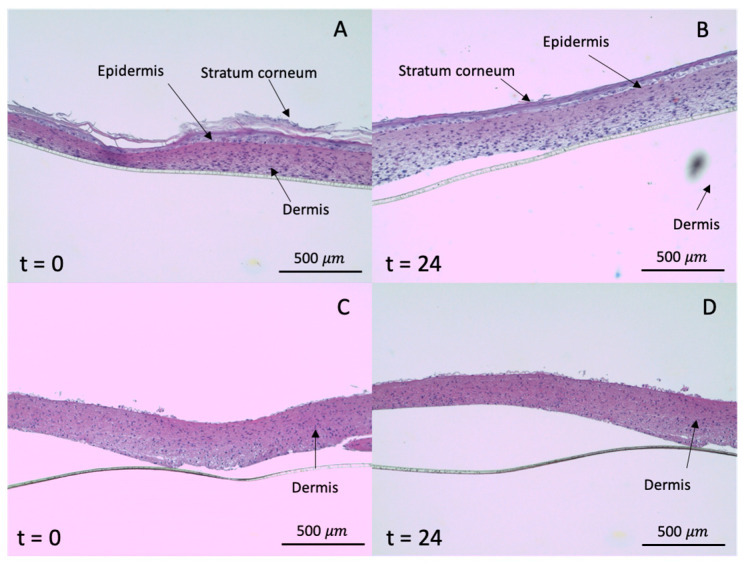
Histological analysis of fixed full thickness skin (containing epidermal and dermal layers, **A** and **B**) and skin equivalents with only dermis (**C** and **D**) before (*t* = 0 h) and after (*t* = 24 h) the LID permeation experiment from GMS-LID4 particles in the cellular inserts.

Histological analysis showed that the skin is not damaged with the presence of the GMS microparticles as all printed skin equivalents maintained their integrity in PBS medium after 24 h of topical treatment (Figure 7B,D). The structures were similar to the skin equivalents initially prepared (Figure 7A,C) with an intact stratum corneum, epidermis, and dermis. Therefore, LID-loaded GMS microparticles have no toxic adverse effects on the skin.

### 3.5. Antimicrobial Activity of the SLMPs

Among the local anesthetics, LID-based formulations are the most studied medications for a supplemental antimicrobial role [9]. The mechanism of antimicrobial action of LID is not fully elucidated but related to the dysfunction of cellular respiration, the alteration of bacterial protein synthesis and the disruption of the bacterial cell wall or cytoplasmic membrane [57,58,59].

**Figure 8:**
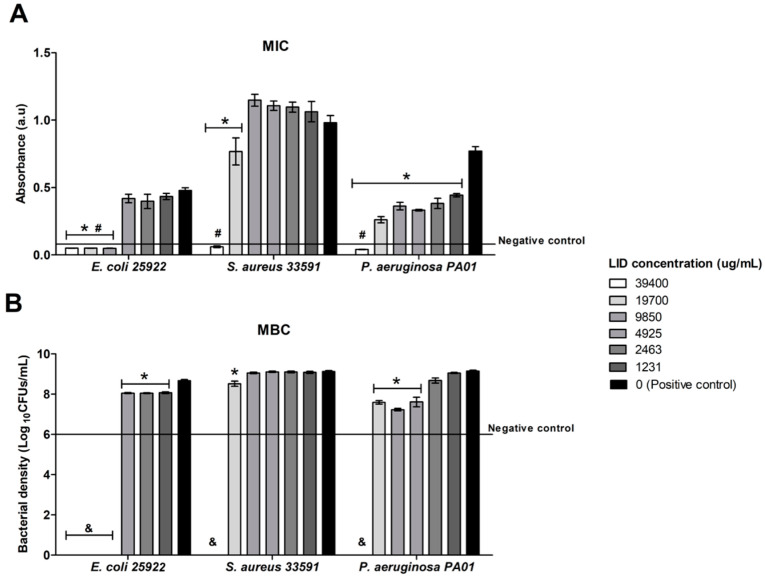
Antimicrobial activity of LID obtained by determination evaluation of minimum inhibitory concentration (MIC) (**A**) and minimum bactericidal concentration (MBC) (**B**) for *Escherichia coli* 25922, *Staphylococcus aureus* 33591, and *Pseudomonas aeruginosa* PA01 strains. * Statistically different (*p* < 0.05) compared with bacterial growth without LID (positive control). ^#^ Absorbance of bacterial growth lower or equal to the control negative (absorbance of medium without bacteria). ^&^ statistically different (*p* < 0.05) compared with initial bacterial concentration (negative control MBC).

The MIC and MBC values for free lidocaine were determined in this work against *E. coli*, *S. aureus,* and *P. aeruginosa* bacterial strains (Figure 8) to define the minimum concentration of LID capable to inhibit the bacterial growth and kill bacteria, respectively. This information is of relevance in this work to define the LID content needed in the SLMP particles to obtain particles with therapeutic potential against the most common bacteria found in infected wounds. MIC is defined as the lowest concentration of antimicrobial agent that inhibits the visible bacterial growth [48]. In order to determine the MIC values, bacteria were cultured in broth containing dilutions of LID and the turbidity of the broths after 24 h of incubation was compared with the turbidity of the negative control (broth without bacteria) [60]. Three concentrations of LID (9850, 19,700, and 39,400 µg/mL) visibly inhibited *E. coli* growth (Figure 8A), whereas only the concentration of 39,400 µg/mL was able to avoid bacterial growth for *S. aureus* and *P. aeruginosa*. Accordingly, MIC for *E. coli* was 9850 µg/mL and for *S. aureus* and *P. aeruginosa* was four-fold higher (39,400 µg/mL). Results correlate well with previous works showing that the LID at low concentrations was more active against *E. coli* strains, whereas for *S. aureus* and *P. aeruginosa* strains higher MICs of LID were needed to avoid their growth [61,62].

MBC is the lowest concentration of antimicrobial agent at which no cultivable bacteria (CFUs) are detected on a solid medium, causing bacterial death. MBC is determined as the lowest concentration of antimicrobial agent that reduces the viability of the initial bacterial inoculum by a predetermined reduction such as ≥99.9% [48]. In this work, the bactericidal activity of LID was tested against the bacterial strains used. The 1 wt.% LID was enough to kill ≥99.9% of *E. coli* bacteria, whereas for *S. aureus* and *P. aeruginosa* a higher LID concentration (ca. 4 wt.%) was needed. Moreover, significant differences were found in terms of bacterial density for all strains compared to a positive control (bacteria without LID) for specific LID concentrations. For instance, for *E. coli*, the concentrations of 0.12, 0.25, and 0.5 wt.% of LID reduced 75% of bacterial density compared with positive control (Figure 8B). For *P. aeruginosa*, this reduction was more pronounced with reductions higher than 95% for LID concentrations of 0.5, 1, and 2 wt.%. These results emphasized the antimicrobial activity of LID [62,63,64], particularly against the most common bacteria found in infected wounds [10].

**Figure 9:**
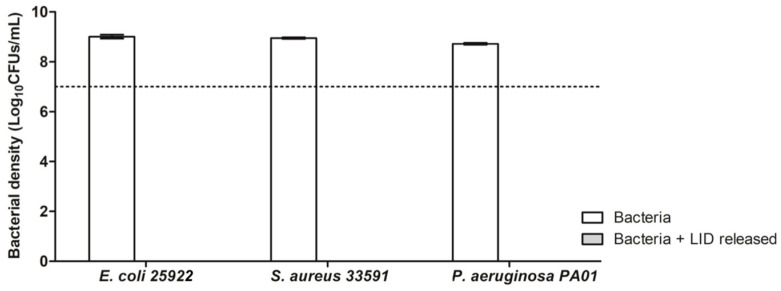
Antimicrobial activity of LID released from GMS-LID10 particles. Bacterial growth without LID after 24 h of incubation was used as control (white bars). Horizontal dashed line indicates the initial bacterial density. The LID released from GMS-LID10 particles was able to kill all bacteria after 24 h of culture.

According to the MIC and MBC values of LID for *E. coli*, *S. aureus,* and *P. aeruginosa* bacteria, GMS-LID10 particles were tested regarding their antimicrobial activity, since the LID concentration levels released from these particles into the medium were higher than the MIC and MBC values for the three bacterial strains. Results from GMS-LID10 particles showed an outstanding antimicrobial activity able to kill all the bacterial strains used after 24 h of incubation (Figure 9). These results further emphasize the therapeutic potential of the developed local delivery approach based on dressings, in the prevention and eradication of relevant bacteria in post-operative wounds, prior to their colonization and biofilm infection establishment.

## 4. Conclusions

GMS microparticles incorporating lidocaine hydrochloride for specific wound delivery applications in pain and infection treatments were prepared by PGSS technique. The obtained particles had a high encapsulation efficiency (70–79%) and a tunable lidocaine content (0.7–7.5 wt.%) that can be adapted to the required doses to get the intended anesthetic and antimicrobial effects. The lidocaine release from the lipid particles fitted to a first-order with lag time kinetic model with 50% of the drug payload released after ca. 4 h. This drug release behavior can result in formulations with reduced doses with respect to commercial formulations with local infiltration of lidocaine. Antimicrobial tests also emphasized the therapeutic potential of the lidocaine-loaded particles in the prevention and full eradication of relevant Gram-positive and Gram-negative bacteria in post-operative wounds, prior to their colonization and biofilm infection establishment. Therefore, the lidocaine content in the formulation can be adjusted to get an attractive anesthetic-plus-antimicrobial combined effect, which is of high relevance for the management of chronic wounds. Diffusion through the skin barrier of lidocaine from the microparticles was evaluated using innovative 3D-bioprinted skin equivalent models mimicking the intact skin and open wound skin tissues. This in vitro test allowed to assess the different permeation behavior of lidocaine depending on the skin condition and failure and to confirm the non-toxicity of the particles for the skin. The full potential of this in vitro skin model needs to be validated and the technology evaluated for the processing of other models for skin under different pathological situations.

## Figures and Tables

**Table 1:** Thermal properties of raw materials and GMS particles processed without (GMS-LID0) and with LID (GMS-LID4).

Thermal Event	LID	GMS	GMS-LID0	GMS-LID4
*T*_m_ (°C)	78.12	62.28	62.46	60.56
Δ*H*_m_ (J/g)	187.0	186.8	179.1	170.9

**Table 2:** Kinetic fitting parameters of LID release profiles from SLMPs in PBS pH 7.4 medium to the first-order and the first-order with lag time kinetic models according to Equations (2) and (3), respectively.

SLMPs	First-Order		First-Order with Lag Time		
	*k*_1_ (h^−1^)	*R* ^2^	*k*_2_ (h^−1^)	*t_lag_* (h)	*R* ^2^
GMS-LID1	0.3047	0.938	0.2532	0.7708	0.966
GMS-LID2	0.2538	0.941	0.2278	0.9049	0.959
GMS-LID4	0.3308	0.963	0.2975	1.066	0.970
GMS-LID10	0.3224	0.985	0.3435	0.4736	0.985

## Supplementary Materials

The following are available online at https://www.mdpi.com/1999-4923/12/9/870/s1, Figure S1. Visual appearance of (A) bioprinted human skin equivalents in cellular inserts, and (B) once removed from the inserts for certain permeation tests in Franz diffusion cells, Figure S2. Release profile (in percentage of drug released) of raw LID (white circles), GMS-LID1 (black circles), GMS-LID2 (diamonds), GMS-LID4 (triangles), and GMS-LID10 (squares) particles in PBS pH 7.4 at 37 °C and 100 rpm.
