# Supplementary material for: Lidocaine-Loaded Solid Lipid Microparticles (SLMPs) Produced from Gas-Saturated Solutions for Wound Applications
Source: Pharmaceutics. 2020 Sep 12;12(9):870. doi: 10.3390/pharmaceutics12090870 (PMC7557821; doi:10.3390/pharmaceutics12090870)
Supplement: Supplementary file 1 [file pharmaceutics-12-00870-s001.pdf]

pharmaceutics

Supplementary Materials: Lidocaine-loaded solid lipid microparticles (SLMPs) produced from gas-saturated solutions for wound applications

Clara López-Iglesias, Cristina Quílez, Joana Barros, Diego Velasco, Carmen Alvarez-Lorenzo, José L. Jorcano, Fernando J. Monteiro and Carlos A. García-González *

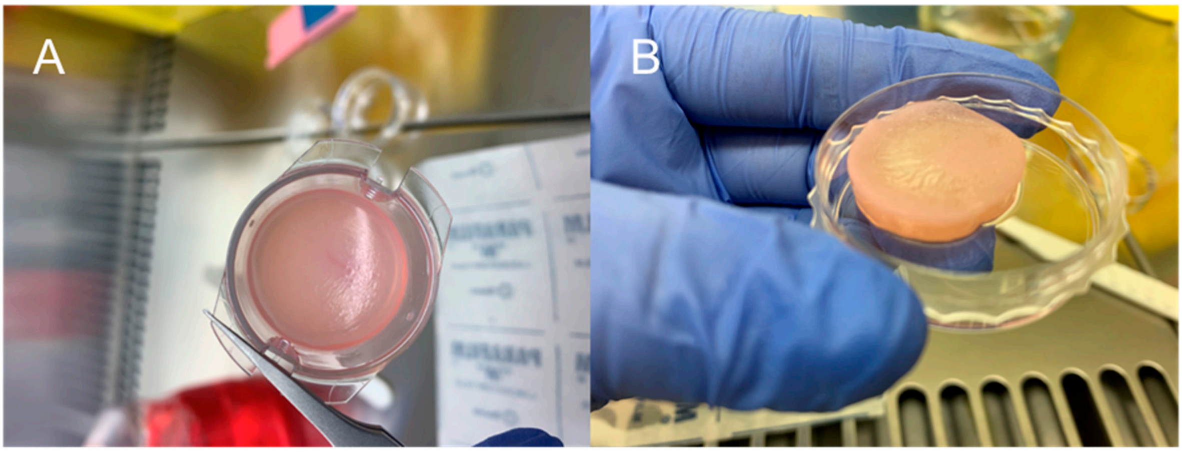

Figure S1. Visual appearance of (A) Bioprinted human skin equivalents in cellular inserts, and (B) once removed from the inserts for certain permeation tests in Franz diffusion cells.

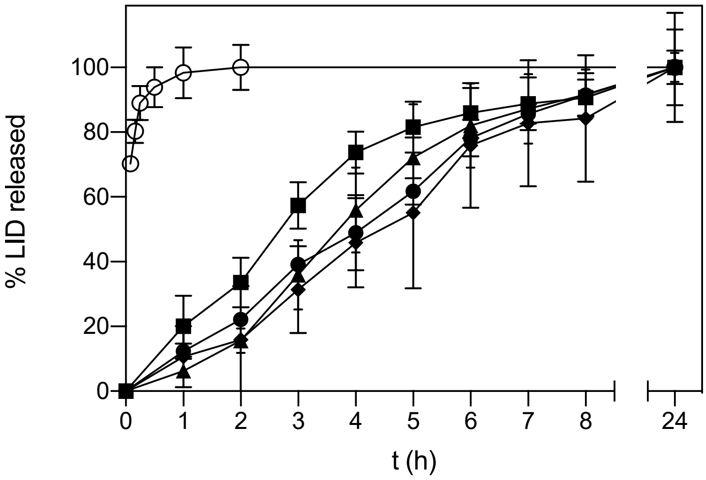

Figure S2. Release profile (in percentage of drug released) of raw LID (white circles), GMS-LID1 (black circles), GMS-LID2 (diamonds), GMS-LID4 (triangles) and GMS-LID10 (squares) particles in PBS pH 7.4 at 37 °C and 100 rpm.
